# Skin Autofluorescence Is Associated with Endothelial Dysfunction in Uremic Subjects on Hemodialysis

**DOI:** 10.1371/journal.pone.0147771

**Published:** 2016-01-25

**Authors:** Chun-Cheng Wang, Yao-Chang Wang, Guei-Jane Wang, Ming-Yi Shen, Yen-Lin Chang, Show-Yih Liou, Hung-Chih Chen, Chiz-Tzung Chang

**Affiliations:** 1 Graduate Institute of Clinical Medical Science, China Medical University, Taichung, Taiwan; 2 Division of Cardiology, Department of Internal Medicine, Taichung Tzuchi Hospital, The Buddhist Tzuchi Medical Foundation, Taichung, Taiwan; 3 Division of Cardiothoracic surgery, Chang Gung Memorial Hospital Keelung Branch, Keelung, Taiwan; 4 Department of Medical Research, China Medical University Hospital, Taichung, Taiwan; 5 Department of Health and Nutrition Biotechnology, Asia University, Taichung, Taiwan; 6 Department of Biomedical Engineering, Chun Yuan Christian University, Taoyuan, Taiwan; 7 Formosan Blood Purification Foundation, Taipei, Taiwan; 8 Division of Nephrology, Department of Internal Medicine, China Medical University Hospital, Taichung, Taiwan; 9 College of Medicine, China Medical University, Taichung, Taiwan; 10 Cardiovascular research laboratory, China Medical University Hospital, Taichung, Taiwan; The University of Manchester, UNITED KINGDOM

## Abstract

**Background:**

Elevated levels of advanced glycation end products (AGEs) within tissues may contribute to endothelial dysfunction, an early indicator of atherosclerosis. We aimed to investigate whether levels of skin AGEs could be a useful marker to predict endothelial dysfunction in uremic subjects on hemodialysis.

**Methods and Results:**

One hundred and nineteen uremic patients on hemodialysis and 57 control subjects with moderate-to-high cardiovascular risk factors and without chronic kidney disease (CKD) were enrolled. We used ultrasound to measure flow-mediated vasodilation (FMD). An AGE reader measured skin autoflurorescence (AF). We then compared differences in FMD and skin AF values between the two groups. The uremic subjects had significantly higher levels of skin AF (3.47±0.76 AU vs. 2.21±0.45 arbitrary units; P<0.01) and significantly lower levels of FMD (4.79%±1.88% vs. 7.19%±2.17%; P<0.01) than the non-CKD subjects. After adjusting for all potential covariates, we found that skin AF level independently predicted FMD in both the hemodialysis and the non-CKD groups. In the hemodialysis group, skin AF ≥ 3.05 arbitrary units predicted abnormal FMD at a sensitivity of 87.9% and a specificity of 78.6% (P<0.01).

**Conclusions:**

Skin AF could be a useful marker to predict endothelial dysfunction in uremic subjects on hemodialysis.

## Introduction

Cardiovascular (CV) disease is one of the major causes of death in uremic patients [1,2], and uremic patients have been reported to have a higher prevalence of conventional CV risk factors than the general population [3]. However, conventional CV risk factors cannot explain accelerated rates of atherosclerotic vascular disease and the equivalent death rates across all age groups seen in uremic patients [4,5]. In uremia, increased inflammation, and increased oxidative stress may contribute to atherosclerosis [5,6].

Advanced glycation end products (AGEs) are formed in a non-enzymatic process called the Maillard reaction, in which reducing sugars modify proteins. The increased oxidative and carbonyl stress may contribute to the formation of AGEs [7–9]. As a result, the accumulation of AGEs is abundant in aging, diabetic, and chronic renal failure patients. The contributory role of AGEs to the pathogenesis of vasculopathy has been reported. AGEs may cross-link with collagen, and elastin within vessel medial layers, thereby increasing vascular stiffness [10]. In addition, AGEs could interact with receptors for AGEs on endothelial cells, further inhibiting endothelial nitric oxide synthase, thereby contributing to endothelial dysfunction [11–13]. Therefore, the AGEs may contribute to both atherosclerosis and arteriosclerosis in patients with uremia.

Although the pathogenic role of AGEs in vasculopathy has been well discussed, the clinical significance of AGEs in uremic patients is controversial [14,15] and whether tissue levels of AGEs could be used to represent vascular dysfunction has yet to be elucidated. Therefore, we conducted this case-control study to compare tissue levels of AGEs and measurements of flow-mediated vasodilatation (FMD) (an indicator of endothelial dysfunction) between uremic subjects on hemodialysis and subjects with non-chronic kidney disease (non-CKD).

## Material and Methods

### Study design and subjects

We prospectively recruited 119 uremic subjects on hemodialysis and compared with 57 subjects with moderate-to-high CV risk factors, and without CKD. The inclusion criteria for the study group included subjects between the ages of 30–80 years at the uremia stage who had received regular hemodialysis as renal replacement therapy for at least 6 months at our outpatient department. None of the uremic subjects enrolled in our study had received peritoneal dialysis or renal transplantation as renal replacement therapy. The inclusion criteria for the non-CKD group included subjects between the ages of 30–80 years who had moderate-to-high CV risk factors, as well as non-CKD status, and who had received regular medications for at least 6 months at our outpatient department. Moderate-to-high CV risk was defined as (1) at least two conventional CV risk factors [family history of premature coronary artery disease, male gender with an age ≥ 45yrs, or female gender with an age ≥ 50yrs, hypertension, diabetes mellitus (DM), hyperlipidemia, and smoking]; (2) a history of ischemic heart disease (IHD), cerebrovascular disease or peripheral arterial occlusive disease (PAOD). Non-CKD was defined as (1) estimated glomerular filtration rate (eGFR) ≥ 60ml/min/1.73m^2^. eGFR was calculated according to the Modification of Diet in Renal Disease (MDRD) Study equation. (2) Absence of functional or structural abnormalities of the kidney. The functional or structural abnormality of the kidney is defined as proteinuria ≥ 30mg/dL estimated by spot urine dipstick method or abnormalities in imaging studies [16]. The exclusion criteria included subjects with dementia, conscious change, a bed-ridden status, an active infection, marked blood pressure fluctuation during hemodialysis, cardiac arrhythmia, history of psychiatric diseases, liver cirrhosis, alcoholism or advanced stage cancer. All of the recruited subjects received both FMD and skin autofluorescence (AF) measurements. In the hemodialysis group, FMD and skin AF were measured in the subjects’ upper extremities that were not used for dialysis during dialysis sessions. In the non-CKD group, FMD and skin AF were measured in the subjects’ right upper extremities. We obtained baseline data and medication history by chart review and patient interview. Data of the subjects’ laboratory tests were all sampled within 3 months of study enrollment. DM was defined as a plasma level of glycohemoglobin ≥ 6.5% or the use of hypoglycemic medications for over 6 months. Hypertension was defined as a series of at least three systolic blood pressure measurements ≥ 140mmHg or diastolic blood pressure measurements ≥ 90mmHg at home or the use of anti-hypertensive medications for over 6 months. Hyperlipidemia was defined as a plasma level of total cholesterol > 200mg/dL, low-density lipoprotein cholesterol > 130mg/dL, triglyceride > 150mg/dL, or the use of lipid-lowering medications for over 6 months. IHD was defined as any evidence of ischemia diagnosed by non-invasive stress testing, or invasive coronary angiography. Cerebrovascular disease was defined as any event of ischemic stroke or transient ischemic attack diagnosed by computed tomography or magnetic resonance imaging. PAOD was defined as an ankle-brachial index (ABI) < 0.9 further confirmed by non-invasive duplex ultrasound, computed tomography, or invasive peripheral angiography. The ethical committees of both the China Medical University hospital and the Taichung Tzuchi hospital approved the study protocol. All patients provided signed informed consent before study enrollment.

### Measurement of FMD

The protocol for the ultrasound assessment of endothelial-dependent FMD has been proposed before [17–19]. The subjects remained in supine position throughout the whole process. An ultrasound Vivid E system (GE Healthcare, Horten, Norway) coupled with a linear array high-resolution transducer (9L-RS; 3.3–10 MHz) was used to measure endothelial-dependent FMD. A sphygmomanometer cuff was placed around the tested forearm, and the brachial artery was scanned about 5 cm above the elbow. The clearest B-mode image of the anterior and posterior intimal layers and vessel wall was obtained, and the transducer was held in place throughout the assessment. The longitudinal image of the brachial artery was acquired, and the baseline diameter of the brachial artery was measured. The diameter of the brachial artery was defined as the distance between the anterior and posterior intimal layers of the brachial artery. The cuff was then inflated up to 250mmHg or at least 50mmHg above the systolic blood pressure for 5 minutes to produce an adequate hyperaemic response. The diameter of the dilated brachial artery was further measured about 45–60 seconds after deflation of the brachial artery. The percentage of change in the brachial artery diameter (percentage of FMD) was used to represent the endothelial function of the brachial artery.

### Measurement of skin AF

Levels of skin AGEs were assessed by measuring skin AF with an AGE reader (DiagnoOptics Technologies BV, Groningen, the Netherlands) [20,21]. An AGE reader is a desktop device that uses the fluorescent properties of some AGEs to estimate AGE accumulation in the skin. The AGE reader illuminated 4 cm^2^ of skin surface guarded against surrounding light with an excitation ultraviolet light with wavelengths between 300–420 nm (peak excitation wavelength of 350 nm). The average light intensity of the emitted light with wavelengths between 420-600nm and reflected excitation light with wavelengths of 300-420nm from the skin were measured with a spectrometer. Skin AF was measured as the ratio of the average light intensity between the emitted light and the reflected excitation light, multiplied by 100, and expressed as arbitrary units (AU). The protocol of skin AF measurement has been described elsewhere [20]. The intra-individual Altman error percentages of repeated skin AF measurements taken over a single day and for seasonal variation were 5.03%, and 5.87%, respectively [20].

### Statistical analysis

The normality assumption for continuous variables were evaluated by the Kolmogorov-Smirnov test. Differences in continuous variables between groups were evaluated using student’s unpaired t test or Mann-Whitney nonparametric tests, as appropriate. Categorical variables were compared with the chi-square test. Pearson’s or Spearman’s correlation tests were performed to investigate the correlations of skin AF, FMD and other variables. Factors with P value < 0.05 were entered into the multiple linear regression analysis to determine the independent factors associated with the FMD value, and the skin AF level. Differences in FMD and AGE levels between the control group, the uremia with DM group, and the uremia without DM group were analyzed using the one-way analysis of variance with post-hoc analysis using Bonferroni’s correction. Receiver operating characteristic (ROC) curve analysis was performed to identify the optimal AGE level that best predicted a FMD value < 6% [22].

## Results

Comparisons of the baseline characteristics between the hemodialysis group and the non-CKD group are shown in Table 1.

**Table 1:** Baseline demographic data of the study groups.

	Non-CKD (n = 57)	Uremia on HD (n = 119)	P value
Age (years)	58.72±10.50	59.60±11.63	0.63
Gender (M/F)	39/18	71/48	0.26
BMI (kg/m2)	27.49±3.73	23.92±3.42	<0.01
Albumin (g/dL)	—	4.15±0.29	—
eGFR (mL/min/1.73m^2^)	72.82[67.43;80.74]	5.70[4.88;6.80]	<0.01
Stroke	5.26%	9.24%	0.36
PAOD	1.75%	11.76%	0.03
IHD	14.04%	38.65%	<0.01
Hyperlipidemia	66.67%	48.73%	0.03
DM	33.33%	45.38%	0.13
HTN	77.19%	82.35%	0.42
CHF	8.77%	15.97%	0.19
hs-CRP (mg/dL)	—	0.34[0.17;0.76]	
Skin AF	2.21±0.45	3.47±0.76	<0.01
FMD	7.19%±2.17%	4.79%±1.88%	<0.01
Medications:			
Antiplatelet	57.89%	45.76%	0.13
β- blockers	43.85%	53.39%	0.23
CCBs	42.10%	54.24%	0.13
Nitrate	24.56%	21.01%	0.60
ACEIs/ARBs	63.15%	37.29%	<0.01
Statin	52.63%	19.49%	<0.01
Insulin	0.00%	22.03%	<0.01
Vintage months	—	70.78±58.26	—

CKD: Chronic kidney disease; HD: Hemodialysis; M: Male; F: Female; BMI: Body mass index; eGFR: estimated glomerular filtration rate; PAOD: Peripheral arterial occlusive disease; IHD: Ischemic heart disease; DM: Diabetes mellitus; HTN: Hypertension; CHF: Congestive heart failure; hs-CRP: high-sensitivity C-reactive protein; AF: Autofluorescence; FMD: Flow-mediated vasodilatation; CCBs: Calcium channel blockers; ACEIs: Angiotensin converting enzyme inhibitors; ARBs: Angiotensin II receptor blockers

The hemodialysis subjects had significantly higher proportions of PAOD, IHD, and a lower proportion of hyperlipidemia than the non-CKD group. The skin AF level was significantly higher (3.47±0.76 AU vs. 2.21±0.45 AU; P<0.01) and the FMD value was significantly lower (4.79%±1.88% vs. 7.19%±2.17%; P<0.01) in hemodialysis subjects compared to the non-CKD group. The hemodialysis subjects had significantly lower proportions of angiotensin converting enzyme inhibitors (ACEIs)/angiotensin II receptor blockers (ARBs), and statins.

Correlations between FMD value and other clinical characteristics were demonstrated in all of the cases, the hemodialysis group, and the non-CKD group (Table 2).

**Table 2:** Univariate correlation analysis between FMD and other covariates in different groups.

	All cases		Uremia with HD		Non-CKD	
	r	P value	r	P value	r	P value
Age	-0.20	<0.01	-0.15	0.11	-0.37	<0.01
Sex	-0.15	0.05	-0.22	0.02	0.07	0.60
BMI	0.38	<0.01	0.15	0.11	0.27	0.04
Albumin	—	—	0.13	0.08	—	—
Stroke	-0.32	0.67	0.03	0.76	-0.06	0.66
PAOD	-0.12	0.10	-0.03	0.76	-0.15	0.27
IHD	-0.20	<0.01	-0.08	0.41	-0.15	0.26
Hyperlipidemia	0.11	0.14	0.03	0.75	0.04	0.79
DM	-0.13	0.86	0.06	0.51	0.03	0.82
HTN	-0.14	0.85	-0.04	0.70	0.11	0.42
CHF	-0.86	0.25	0.04	0.66	-0.25	0.06
hs-CRP	—	—	-0.23	0.07	—	—
Skin AF	-0.79	<0.01	-0.80	<0.01	-0.46	<0.01
ESRD	-0.50	<0.01	—	—	—	—
Antiplatelet	0.11	0.16	0.06	0.50	0.55	0.68
β-blockers	-0.20	0.79	0.06	0.53	-0.03	0.84
CCBs	0.01	0.91	0.10	0.26	0.02	0.86
Nitrate	0.08	0.27	0.01	0.88	0.18	0.18
ACEI/ARB	0.19	0.01	0.10	0.29	0.06	0.65
Statin	0.24	<0.01	0.11	0.23	0.04	0.75
Insulin	-0.14	0.06	0.00	0.97	—	—
Vintage months	—	—	-0.20	0.03	—	—

HD: Hemodialysis, CKD: Chronic kidney disease; FMD: Flow-mediated vasodilatation; BMI: Body mass index; PAOD: Peripheral arterial occlusive disease; IHD: Ischemic heart disease; DM: Diabetes mellitus; HTN: Hypertension; CHF: Congestive heart failure; hs-CRP: high-sensitivity C-reactive proteon; AF: Autofluorescence; ESRD: end-stage renal disease; CCBs: Calcium channel blockers; ACEIs: Angiotensin converting enzyme inhibitors; ARBs: Angiotensin II receptor blockers

In all of the cases, aging, a lower body mass index (BMI), the presence of IHD, higher skin AF level, uremic stage on hemodialysis, and no statin use were associated with a lower FMD value. In the hemodialysis group, female gender, higher skin AF level, and longer duration of dialysis were associated with a lower FMD value. In the non-CKD group, aging, a lower body mass index, and higher skin AF level were associated with a lower FMD value.

After adjusting for all potential covariates, the skin AF level was an independent predictor for FMD value in all subjects, the hemodialysis group, and the non-CKD group (Table 3).

**Table 3:** Multivariate linear regression analysis for the association between skin AF and FMD in (a) all cases, (b) uremia on hemodialysis group, and (c) non-chronic kidney disease group.

(a)				
Variables	Unstandardized Coefficient B	SE	Standardized Coefficient β	P value
AF	-1.66	0.19	-0.65	<0.01
Age	-0.01	0.01	-0.05	0.38
BMI	0.44	0.04	0.08	0.22
IHD	-0.21	0.28	-0.04	0.46
Uremia	0.10	0.38	0.02	0.79
Statin	0.47	0.29	0.10	0.11
ACEIs/ARBs	0.18	0.25	0.04	0.47
(b)				
Variables	Unstandardized Coefficient B	SE	Standardized Coefficient β	P value
AF	-1.60	0.18	-0.65	<0.01
Gender	-0.38	0.27	-0.10	0.16
Vintage months	-0.00	0.00	-0.02	0.82
(c)				
Variables	Unstandardized Coefficient B	SE	Standardized Coefficient β	P value
AF	-1.85	0.66	-0.38	<0.01
Age	-0.03	0.03	-0.13	0.35
BMI	0.10	0.07	0.17	0.16

AF: Autofluorescence; FMD: Flow mediated vasodilatation; SE: standard error; BMI: Body mass index; IHD: Ischemic heart disease; ACEIs: Angiotensin converting enzyme inhibitors; ARBs: Angiotensin II receptor blockers.

**Fig 1:**
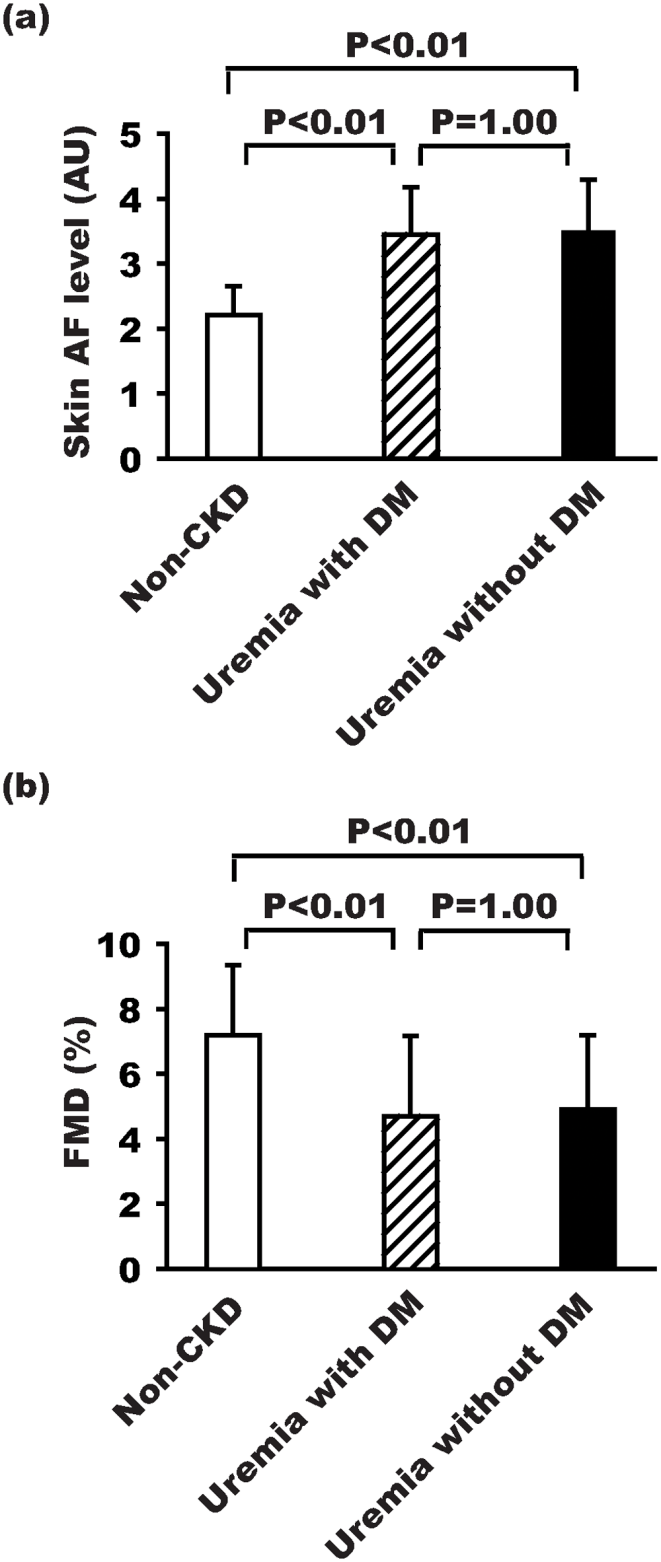
Using one-way analysis of variance and post-hoc multiple comparisons with Bonferroni’s correction to investigate the differences of skin AF levels, and FMD values between the non-CKD, uremia with DM, and uremia without DM groups. (a) The skin AF level was significantly lower in the non-CKD group than in the uremia group (P<0.01). However, the skin AF level was similar between the uremia with or without DM groups. (b) The FMD value was significantly higher in the non-CKD group than in the uremia group (P<0.01). However, the FMD value was similar between the uremia with or without DM groups.

Differences in skin AF levels and FMD values between the non-CKD group, the uremia with DM group, and the uremia without DM group were compared (Fig 1). The skin AF levels and FMD values were similar between the uremia with DM and uremia without DM groups. The skin AF levels in these two groups were significantly higher than that of the non-CKD group, and the FMD values in these two groups were significantly lower than that of the non-CKD group.

Comparisons of the baseline characteristics of the uremia with DM group and the uremia without DM group are shown in Table 4.

**Table 4:** Comparisons of baseline characteristics between the uremia with DM and the uremia without DM groups.

	Uremia with DM (n = 54)	Uremia without DM (n = 65)	P-value
Age (years)	62.13 ± 9.57	57.49 ± 12.78	0.03
Gender (M/F)	33/21	38/27	0.77
BMI (kg/m2)	24.67 ± 3.38	23.29 ± 3.36	0.03
Stroke	9.26%	9.23%	1.00
PAOD	20.37%	4.62%	0.01
IHD	50.00%	29.23%	0.02
Hyperlipidemia	62.96%	36.92%	<0.01
DM	100.00%	—	—
HTN	88.89%	76.92%	0.09
CHF	20.37%	12.31%	0.23
AF	3.50 ± 7.92	3.44 ± 7.38	0.69
FMD	4.90 ± 2.28	4.69 ± 1.48	0.56
Medications:			
Antiplatelet	58.49%	35.38%	0.01
β - blockers	64.15%	44.62%	0.03
CCBs	56.60%	52.31%	0.64
Nitrate	27.78%	15.38%	0.10
ACEIs/ARBs	45.28%	30.77%	0.11
Statin	28.30%	12.31%	0.03
Insulin	49.06%	0.00%	—
Vintage months	47.63 ± 36.49	90.31 ± 65.85	<0.01

DM: Diabetes mellitus; BMI: Body mass index; PAOD: Peripheral arterial occlusive disease; IHD: ischemic heart disease; HTN: Hypertension; CHF: Congestive heart failure; AF: Autofluorescence; FMD: Flow mediated vasodilatation; CCBs: Calcium channel blockers; ACEIs: Angiotensin converting enzyme inhibitors; ARBs: Angiotensin II receptor blockers.

Compared to the uremia without DM group, the uremia with DM group had a significantly older age, higher body mass index, and higher proportions of PAOD, IHD, and hyperlipidemia. The uremia with DM group had a significantly higher use of antiplatelets, β –blockers, and statins.

Correlations between skin AF and other covariates in all cases were assessed and the result is demonstrated in S1 Table. Uremic stage on hemodialysis, age, gender, BMI, PAOD, IHD, DM, FMD, medications with ACEIs/ARBs, and statins, were significantly correlated with skin AF. After adjustment for all potential covariates, uremic stage on hemodialysis is still an independent factor associated with skin AF level (Table 5).

**Table 5:** Multivariate linear regression analysis to investigate independent factors associated with skin AF in all cases.

	Unstandardized Coefficient B	SE	Standardized Coefficient β	P value
Uremia with HD	0.72	0.12	0.38	<0.01
Age	0.01	0.004	0.06	0.21
Gender	0.10	0.09	0.06	0.21
BMI	-0.004	0.12	-0.02	0.72
PAOD	0.41	0.15	0.13	<0.01
IHD	0.03	0.10	0.01	0.77
ACEIs/ARBs	-0.03	0.08	-0.02	0.75
Statin	0.02	0.10	0.01	0.86
DM	0.03	0.06	0.02	0.68
FMD	-0.19	0.02	-0.48	<0.01

HD: Hemodialysis; AF: Autofluoresence; SE: Standard error; BMI: Body mass index; PAOD: Peripheral arterial occlusive disease; IHD: Ischemic heart disease; ACEIs: Angiotensin- converting enzyme inhibitors; ARBs: Angiotensin II receptor blockers; DM: Diabetes mellitus; FMD: Flow-mediated vasodilatation.

**Fig 2:**
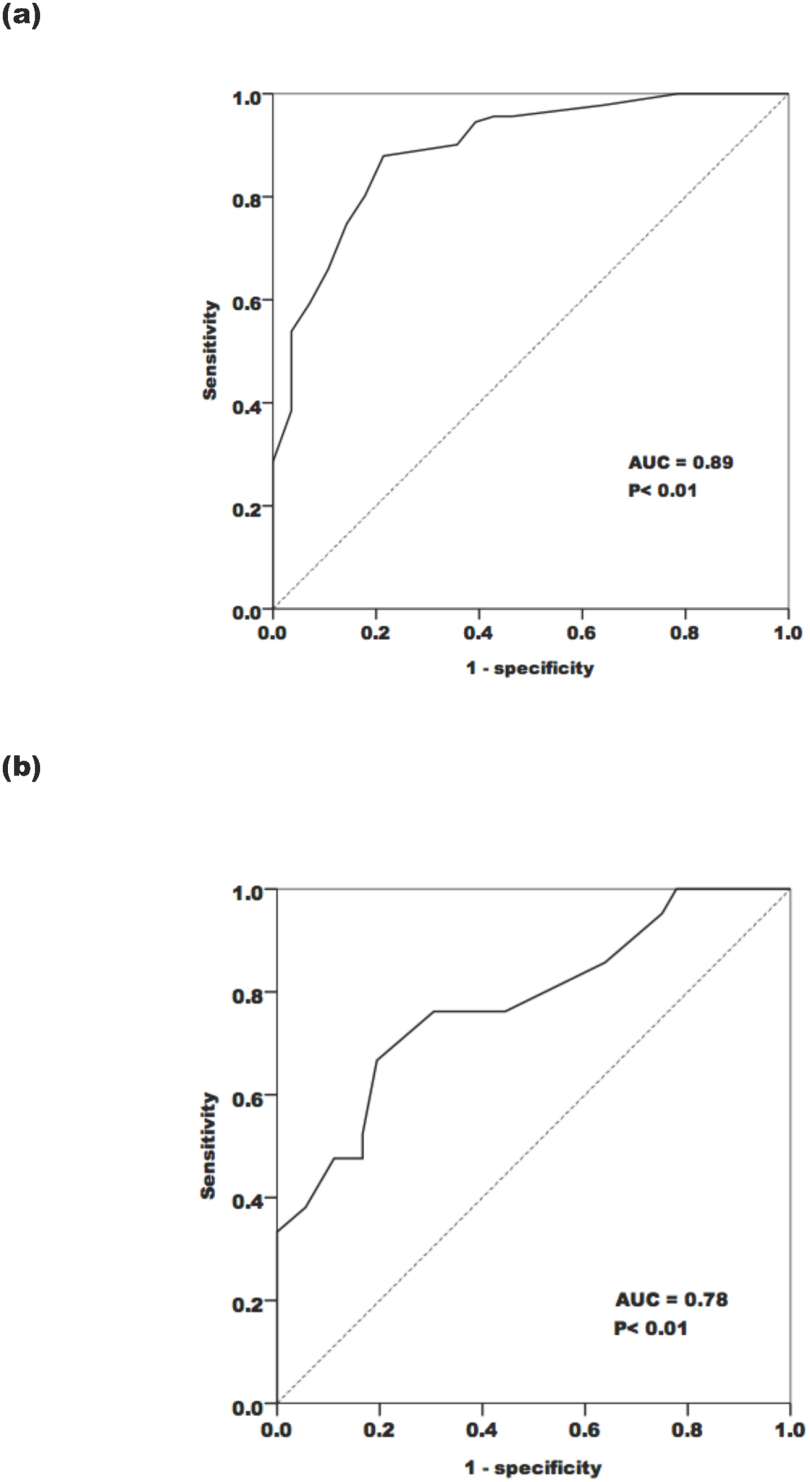
Receiver operating curves (ROC) of AGE for predicting abnormal FMD in the (a) hemodialysis group, and (b) the non-CKD group.

The optimal cut-off value of skin AGE level to determine an abnormal FMD value (FMD < 6%) is shown in Fig 2. In the hemodialysis group, skin AF ≥ 3.05 AU predicted FMD < 6% at a sensitivity of 87.9% and a specificity of 78.6%, with an area under the curve (AUC) of 0.89 (95% CI: 0.83–0.96; P< 0.01). In the non-CKD group, skin AF ≥ 2.25 AU predicted FMD < 6% at a sensitivity of 66.7% and a specificity of 80.6% with an AUC of 0.78 (95% CI: 0.65–0.91; P< 0.01).

## Discussion

Our results provided evidence that measurements of skin AF could be a useful marker to predict endothelial dysfunction in uremic subjects on hemodialysis.

### Clinical applications of skin AF

Previous studies have suggested that skin AGE accumulation could be used to predict both microvascular and macrovascular complications in DM patients [23–25]. The clinical application of skin AF in predicting arterial atherosclerotic disease has also been investigated [26–28]. Although the clinical application of skin AF in predicting cardiovascular outcomes has been widely explored, studies of the clinical significance of skin AF in the setting of uremia are less common. Previous studies have reported that skin AF was an independent predictor for vascular stiffness in patients with uremia [29]. To the best of our knowledge, whether skin AF can be used as a marker for endothelial dysfunction, an indicator for early atherosclerosis, has yet to be clearly defined. Kocak et al. evaluated the role of oxidative stress in predicting endothelial dysfunction in peritoneal dialysis patients, and concluded that serum advanced oxidative protein products, rather than serum AGEs, could predict endothelial dysfunction [30]. However, levels of plasma AGEs may not reflect the tissue content of AGEs, and therefore we used skin AF level instead as a proxy for the tissue content of AGEs. Our results suggest that skin AF level could be used as a marker for endothelial dysfunction in the setting of uremic stage on hemodialysis.

### Associations between AGEs, uremia and endothelial dysfunction

In our study, the baseline characteristics are quite different between the hemodialysis and the non-CKD groups. Therefore, it is not clear whether the significantly elevated skin AF level in the uremia group is due to renal failure itself or because of underlying comorbidities. After adjustment for all potential covariates, uremic stage on hemodialysis is still independently associated with elevated skin AF level. Our data suggest that renal failure itself may contribute to increased tissue AGE accumulation. In addition, we demonstrated that the hemodialysis group has significantly higher skin AF level, and significantly lower FMD value than the non-CKD group. Skin AF level is an independent factor associated with FMD value in both the hemodialysis and the non-CKD groups. Considering all these results, tissue AGE accumulation is associated with endothelial dysfunction in the setting of uremic stage on hemodialysis. Measurement of tissue AGE accumulation may be used as a surrogate marker for endothelial dysfunction in uremic subjects on hemodialysis.

### Associations between ACEIs/ARBs, statin use and endothelial dysfunction

The patients who used ACEIs/ARBs, or statins had better FMD values than the patients who did not use ACEIs/ARBs or statins (Table 2). Previous studies have suggested that statins can upregulate endothelial nitric oxide synthase, thereby improving endothelial-dependent vasodilatation of rat aorta [31,32]. The interaction between angiotensin II and angiotensin II receptor type I may increase reactive oxygen species production, further attenuating the nitric oxide and bradykinin production. Therefore, blockade of the renin-angiotensin system with ACEIs/ARBs may improve endothelial dysfunction.

### Association between diabetes and AGEs accumulation in the setting of uremia

Whether diabetes is associated with increased tissue content of AGEs in the setting of uremia is still controversial. Nazratun et al. obtained arterial and venous biopsy specimens from patients with DM or non-DM associated renal failure and healthy subjects. They found that higher proportions of vessels from the patients with DM related renal failure were positively stained for AGEs than those from non-DM related renal failure or healthy subjects [33]. However, in other studies, serum AGEs levels were similar between uremic subjects with or without diabetes [8,14,34]. Our results suggest that tissue AGEs deposition was similar between the uremic subjects with or without diabetes. This implies that the increased AGEs accumulation in subjects with uremia may be more due to increased oxidative stress, rather than increased glucose burden. However, the uremia with DM group received significantly more antiplatelet therapy and had a significantly shorter duration of dialysis compared to the uremia without DM group. Aspirin has been proposed to decrease the skin pentosidine level in DM patients [35]. In addition, protein-bound AGEs cannot be filtered through a hemodialysis membrane [36]. Patients with a longer duration of dialysis may therefore tend to have a higher accumulation of AGEs. These factors may have caused bias in our results.

### Study limitation

First, as this is a case-control study, we could only establish an association, not a causal effect, between skin AGEs levels and abnormal FMD values. Whether treatment with AGE breaker or inhibition of AGE formation could reverse the endothelial dysfunction in uremic subjects required further investigation. However, the major merit in our study is that we provide a simple, useful method for evaluation of endothelial dysfunction, especially in the setting of uremic stage on hemodialysis. Second, inflammation plays an important role in the pathogenesis of endothelial dysfunction, and has been positively associated with an increased accumulation of AGEs [37]. We could only provide plasma levels of hs-CRP in the hemodialysis group. We could not provide plasma levels of other inflammatory markers; hence, the independent role of tissue AGE accumulation in relation to abnormal FMD in our study may be confounded by other potential inflammatory markers. Third, the study result cannot be extrapolated to subjects aged < 30years, or > 80years. Fourth, we only enrolled uremic subjects on hemodialysis, and those of non-CKD. As the aim of the study was to observe the relation between tissue AGE accumulation and FMD in hemodialysis subjects, the study results could be extrapolated to neither CKD subjects, nor uremic subjects on peritoneal dialysis or renal transplantation as renal replacement therapy. Fifth, skin AF can be used to detect AGEs with fluorescent properties, however, non-fluorescent AGEs cannot be detected by skin AF. Nevertheless, previous studies have reported that skin AF levels were positively correlated with the amount of skin accumulation of both fluorescent and non-fluorescent AGEs [20]. Finally, we used skin AGEs deposition as a proxy for tissue accumulation of AGEs. Further studies are needed to elucidate whether skin AGE deposition can reflect the vascular deposition of AGEs.

## Conclusion

Skin AF is a useful marker to predict endothelial dysfunction in hemodialysis subjects. A skin AF level ≥ 3.05 AU could predict an abnormal FMD response (FMD < 6%) in the hemodialysis subjects. This non-invasive, convenient, and replicable method can be used to detect endothelial dysfunction in the setting of uremic stage on hemodialysis.

## Supporting Information

S1 Table Univariate correlation analysis between skin autofluorescence and other covariates in all cases. (PDF)

## References

[1] Shafi T, Meyer TW, Hostetter TH, Melamed ML, Parekh RS, Hwang S, et al Free levels of selected organic solutes and cardiovascular morbidity and mortality in hemodialysis patients: Results from retained organic solutes and clinical outcomes (ROSCO) investigators. PLoS One 2015; 10(5): e0126048 10.1371/journal.pone.0126048 25938230 PMC4418712

[2] U.S. Renal Data system, USRDS 2013 Annual Data Report: Atlas of chronic kidney disease and end stage renal disease in the United States, National Institute of Health, National Institute of Diabetes and Digestive and Kidney Diseases, Bethesda, Maryland. 2015; 4: 2.

[3] Parfrey PS, Foley RN. The clinical epidemiology of cardiac disease in chronic renal failure. J Am Soc Nephrol 1999; 10: 1606–1615. 10405218 10.1681/ASN.V1071606

[4] Foley RN, Murray AM, Li S, Herzog CA, McBean AM, Eggers PW, et al Chronic kidney disease and the risk for cardiovascular disease, renal replacement, and death in the united states medicare population, 1998–1999. J Am Soc Nephrol 2005; 16: 489–495. 15590763 10.1681/ASN.2004030203

[5] Himmelfarb J, Stenvinkel P, Ikizler TA, Hakim RM. The elephant in uremia: oxidant stress as a unifying concept of cardiovascular disease in uremia. Kidney Int 2002; 62: 1524–1538. 12371953 10.1046/j.1523-1755.2002.00600.x

[6] Widlansky ME, Gokce N, Keaney JF, Vita JA. The clinical implications of endothelial dysfunction. J Am Coll Cardiol 2003; 42: 1149–1160. 14522472 10.1016/s0735-1097(03)00994-x

[7] Miyazawa T, Nakagawa K, Shimasaki S, Nagai R. Lipid glycation and protein glycation in diabetes and atherosclerosis. Amino Acids 2012; 42(4): 1163–1170. 10.1007/s00726-010-0772-3 20957396

[8] Weiss MF, Erhard P, Kader-Attia FA, Wu YC, Deoreo PB, Araki A, et al Mechanisms for the formation of glycoxidation products in end-stage renal disease. Kidney Int 2000; 57: 2571–2585. 10844627 10.1046/j.1523-1755.2000.00117.x

[9] Miyata T, Saito A, Kurokawa K, de Strihou CVY. Advanced glycation and lipoxidation end products: reactive carbonyl compounds-related uraemic toxicity. Nephrol Dial Transplant 2001; 16(suppl 4): 8–11. 11402090 10.1093/ndt/16.suppl_4.8

[10] Sakata N, Noma A, Yamamoto Y, Okamoto K, Meng J, Takebayashi S, et al Modification of elastin by pentosidine is associated with the calcification of aortic media in patients with end-stage renal disease. Nephrol Dial Transplant 2003; 18: 1601–1609. 12897101 10.1093/ndt/gfg200

[11] Brunet P, Gondouin B, Duval-Sabatier A, Dou L, Cerini C, Dignat-George F, et al Does uremia cause vascular dysfunction? Kidney Blood Press Res 2011; 34: 284–290. 10.1159/000327131 21691132

[12] Soro-Paavonen A, Zhang WZ, Venardos K, Coughlan MT, Harris E, Tong DC, et al Advanced glycation end-products induce vascular dysfunction via resistance to nitric oxide and suppression of endothelial nitric oxide synthase. J Hypertens 2010; 28: 780–788. 10.1097/HJH.0b013e328335043e 20186099

[13] Raj DSC, Choudhury D, Welbourne TC, Levi M. Advanced glycation end products: A nephrologist’s perspective. Am J Kidney Dis 2000; 35(3): 365–80. 10692262 10.1016/s0272-6386(00)70189-2

[14] Wagner Z, Molnár M, Molnár GA, Tamaskó M, Laczy B, Wagner L et al Serum carboxymethyllysine predicts mortality in hemodialysis patients. Am J Kidney Dis 2006; 47(2): 294–300. 16431258 10.1053/j.ajkd.2005.10.010

[15] Schwedler SB, Metzger T, Schinzel R, Wanner C. Advanced glycation end products and mortality in hemodialysis patients. Kidney Int 2002; 62: 301–310. 12081592 10.1046/j.1523-1755.2002.00423.x

[16] National Kidney Foundation. K/DOQI clinical practice guidelines for chronic kidney disease: evaluation, classification, and stratification. Am J Kidney Dis 2002;39:S1–S226. 11904577

[17] Shechter M, Issachar A, Marai I, Koren-Morag N, Freinark D, Shahar Y, et al Long-term association of brachial artery flow-mediated vasodilatation and cardiovascular events in middle-aged subjects with no apparent heart disease. Int J Cardiol 2009; 134: 52–58. 10.1016/j.ijcard.2008.01.021 18479768

[18] Raitakari OT, Celermajer DS. Flow-mediated dilatation. Br J Clin Pharmacol 2000; 50: 397–404. 11069434 10.1046/j.1365-2125.2000.00277.x PMC2014404

[19] Iwamoto Y, Maruhashi T, Fujii Y, Idei N, Fujimura N, Mikami S, et al Intimal-media thickness of brachial artery, vascular function, and cardiovascular risk factors. Arterioscler Thromb Vasc Biol 2012; 32: 2295–2303. 10.1161/ATVBAHA.112.249680 22796580

[20] Meerwaldt R, Graaff R, Oomen PHN, Links TP, Jager JJ, Alderson NL, et al Simple non-invasive assessment of advanced glycation end product accumulation. Diabetologia 2004; 47: 1324–330. 15243705 10.1007/s00125-004-1451-2

[21] de Vos LC, Noordzij MJ, Mulder DJ, Smit AJ, Lutgers HL, Dullaart RP, et al Skin autofluorescence as a measure of advanced glycation end product deposition is elevated in peripheral artery disease. Arterioscler Thromb Vasc Biol 2013; 33(1): 131–138. 10.1161/ATVBAHA.112.300016 23139292

[22] Teragawa H, Kato M, Kurokawa J, Yamagata T, Matsuura H, Chayama K. Usefulness of flow-mediated dilation of the brachial artery and/or the intimal-media thickness of the carotid artery in predicting coronary narrowing in patients suspected of having coronary artery disease. Am J Cardiol 2001; 15: 88(10): 1147–1151. 11703961 10.1016/s0002-9149(01)02051-3

[23] Monnier VM, Bautista O, Kenny D, Sell DR, Fogarty J, Dahms W, et al skin collagen ancillary study group. Skin collagen glycation, glycoxidation, and crosslinking are lower in subjects with long-term intensive versus conventional therapy of type 1 diabetes. Diabetes 1999; 48(4): 870–880. 10102706 10.2337/diabetes.48.4.870 PMC2862597

[24] Monnier VM, Sell DR, Genuth S. Glycation products as markers and predictors of the progression of diabetic complications. Ann N Y Acad Sci 2005; 1043: 567–581. 16037280 10.1196/annals.1333.065

[25] Tanaka K, Tani Y, Asai J, Nemoto F, Kusano Y, Suzuki H, et al Skin autofluorescence is associated with severity of vascular complications in Japanese patients with type 2 diabetes. Diabet Med 2012; 29: 492–500. 10.1111/j.1464-5491.2011.03448.x 21916970

[26] Mulder DJ, van Haelst PL, Graaff R, Gans RO, Zijlstra F, Smit AJ. Skin autofluorescence is elevated in acute myocardial infarction and is associated with the one-year incidence of major adverse cardiac events. Neth Heart J 2009; 17(4): 162–168. 19421362 10.1007/BF03086239 PMC2669246

[27] Mulder DJ, van Haelst PL, Gross S, de Leeuw K, Bijzet J, Graaff R, et al Skin autofluorescence is elevated in patients with stable coronary artery disease and is associated with serum levels of neopterin and the soluble receptor for advanced glycation end products. Atherosclerosis 2008; 197(1): 217–223. 17499742 10.1016/j.atherosclerosis.2007.03.027

[28] Noordzij MJ, Lefrandt JD, Loeffen EA, Saleem BR, Meerwaldt R, Lutgers HL et al Skin autofluorescence is increased in patients with carotid artery stenosis and peripheral artery disease. Int J Cardiovasc Imaging 2012; 28(2): 431–438. 10.1007/s10554-011-9805-6 21336554 PMC3288376

[29] Ueno H, Koyama H, Tanaka S, Fukumoto S, Shinohara K, Shoji T, et al Skin autofluorescence, marker for advanced glycation end product accumulation, is associated with arterial stiffness in patients with end-stage renal disease. Metabolism 2008; 57(10): 1452–1457. 10.1016/j.metabol.2008.05.016 18803952

[30] Kocak H, Gumuslu S, Sahin E, Ceken K, Gocmen YA, Yakupoglu G, et al Advanced oxidative protein products are independently associated with endothelial function in peritoneal dialysis patients. Nephrology 2009; 14: 273–280. 10.1111/j.1440-1797.2008.01062.x 19076287

[31] Sönmez Uydeş-Doğan B, Topal G, Takir S, Ilkay Alp F, Kaleli D, Ozdemir O. Relaxant effects of pravastatin, atorvastatin, and cerivastatin on isolated rat aortic rings. Life Sci 2005; 76(15): 1771–1786. 15698855 10.1016/j.lfs.2004.11.002

[32] Deng HF, Xiong Y. Effect of pravastatin on impaired endothelium-dependent relaxation induced by lysophosphatidylcholine in rat aorta. Acta Pharmacol Sin 2005; 26(1): 92–98. 15659120 10.1111/j.1745-7254.2005.00013.x

[33] Nazratun N, Mahmood AA, Kuppusamy UR, Ahmad TS, Tan SY. Diabetes mellitus exacerbates advanced glycation end product accumulation in the veins of end-stage renal failure patients. Vasc Med 2006; 11: 245–250. 17390548 10.1177/1358863x06072202

[34] Zhou Y, Yu Z, Jia H, Sun F, Ma L, Guo R, et al Association of serum pentosidine with arterial stiffness in hemodialysis patients. Artif Organs 2010; 34(3): 193–199. 10.1111/j.1525-1594.2009.00801.x 20447043

[35] Contreras I, Reiser KM, Martinez N, Giansante E, Lopez T, Suarez N, et al Effects of aspirin or basic amino acids on collagen cross-links and complications in NIDDM. Diabetes Care 1997; 20(5): 832–835. 9135951 10.2337/diacare.20.5.832

[36] Jadoul M, Ueda Y, Yasuda Y, Saito A, Robert A, Ishida N, et al Influence of hemodialysis membrane type on pentosidine plasma level, a marker of carbonyl stress. Kidney Int 1999; 55: 2487–2492. 10354298 10.1046/j.1523-1755.1999.00468.x

[37] Suliman ME, Heimbürger O, Bárány P, Anderstam B, Pecoits-Filho R, Rodríguez Ayala E, et al Plasma pentosidine is associated with inflammation and malnutrition in end-stage renal disease patients starting on dialysis therapy. J Am Soc Nephrol 2003; 14(6): 1614–1622. 12761263 10.1097/01.asn.0000067413.32377.cf

