# Supplementary material for: Skin Autofluorescence Is Associated with Endothelial Dysfunction in Uremic Subjects on Hemodialysis
Source: PLoS One. 2016 Jan 25;11(1):e0147771. doi: 10.1371/journal.pone.0147771 (PMC4726548; doi:10.1371/journal.pone.0147771)
Supplement: S1 Table — (PDF) [file pone.0147771.s001.pdf]

	skin AF	
	r	P value
Uremia on HD	0.70	<0.01
Age	0.19	0.01
Gender (F vs M)	0.17	0.02
BMI	-0.37	<0.01
CHF	0.14	0.07
Stroke	0.11	0.14
PAOD	0.24	<0.01
IHD	0.26	<0.01
Hyperlipidemia	-0.02	0.84
HTN	-0.01	0.92
DM	0.17	0.02
Antiplatelets	-0.05	0.51
β-blockers	0.05	0.54
CCBs	0.01	0.90
Nitrate	-0.06	0.45
ACEIs/ARBs	-0.22	<0.01
Statin	-0.22	<0.01
FMD	-0.72	<0.01

HD: Hemodialysis; AF: Autofluorescence; F: Female; M: Male; BMI: Body mass index; CHF: Congestive heart failure; PAOD: Peripheral arterial occlusive disease; IHD Ischemic heart disease; HTN: Hypertension; DM: Diabetes mellitus; CCBs: Calcium channel blockers; ACEIs: Angiotensin-converting enzyme inhibitors; ARBs: Angiotensin II receptor blockers; FMD: Flow-mediated vasodilatation.
